# Functional prediction of long non-coding RNAs in ovarian cancer-associated fibroblasts indicate a potential role in metastasis

**DOI:** 10.1038/s41598-017-10869-y

**Published:** 2017-09-04

**Authors:** Fatemeh Vafaee, Emily K. Colvin, Samuel C. Mok, Viive M. Howell, Goli Samimi

**Affiliations:** 1 School of Biotechnology and Biomolecular Sciences, University of New South Wales, Sydney, NSW 2052 Australia; 2 Bill Walsh Translational Cancer Research Laboratory, Kolling Institute, Northern Sydney Local Health District, St Leonards, NSW 2065 Australia; 3 Sydney Medical School Northern, University of Sydney, Sydney, NSW 2006 Australia; 4 Department of Gynecologic Oncology and Reproductive Medicine Research, Division of Surgery, The University of Texas MD Anderson Cancer Center, Houston, TX USA; 5 Division of Cancer Prevention, National Cancer Institute, National Institutes of Health, Bethesda, MD United States

## Abstract

Cancer-associated fibroblasts (CAFs) contribute to the poor prognosis of ovarian cancer. Unlike in tumour cells, DNA mutations are rare in CAFs, raising the likelihood of other mechanisms that regulate gene expression such as long non-coding RNAs (lncRNAs). We aimed to identify lncRNAs that contribute to the tumour-promoting phenotype of CAFs. RNA expression from 67 ovarian CAF samples and 10 normal ovarian fibroblast (NOF) samples were analysed to identify differentially expressed lncRNAs and a functional network was constructed to predict those CAF-specific lncRNAs involved in metastasis. Of the 1,970 lncRNAs available for analysis on the gene expression array used, 39 unique lncRNAs were identified as differentially expressed in CAFs *versus* NOFs. The predictive power of differentially expressed lncRNAs in distinguishing CAFs from NOFs were assessed using multiple multivariate models. Interrogation of known transcription factor-lncRNA interactions, transcription factor-gene interactions and construction of a context-specific interaction network identified multiple lncRNAs predicted to play a role in metastasis. We have identified novel lncRNAs in ovarian cancer that are differentially expressed in CAFs compared to NOFs and are predicted to contribute to the metastasis-promoting phenotype of CAFs.

## Introduction

Ovarian cancer is the most lethal gynaecologic malignancy in women, of which high-grade serous ovarian cancer (HGSOC) is the most common and aggressive subtype^1^. The majority of women are diagnosed at an advanced stage when the tumour has metastasised throughout the peritoneal cavity, and while initial response to the currently used chemotherapies is high, the development of chemoresistance is common^2^. As such, the 5-year survival rate is reduced from 80% if diagnosed early to <30% for women diagnosed with advanced disease^3^. A greater understanding of the factors that promote metastasis and chemoresistance may improve the ability to provide new and more effective therapeutic targets for the treatment of HGSOC.

The tumour microenvironment is now established as an essential part of tumour progression^4^. Cancer-associated fibroblasts (CAFs) are the most abundant cell type in the tumour microenvironment and are responsible for the secretion of extracellular matrix components, growth and differentiation factors. It has long been acknowledged in multiple cancer types that CAFs interact with tumour cells and play a vital role in promoting cancer cell proliferation, migration and invasion, as well as contribute to angiogenesis and chemoresistance^5–9^. CAFs are also recognised to play a similar role in ovarian cancer^10–13^. Therefore, understanding the molecular characteristics of ovarian CAFs compared to normal ovarian fibroblasts (NOFs) is pivotal in designing effective therapies that target the tumour microenvironment.

Expression profiling studies have identified differential expression of several genes in CAFs or tumour-associated stroma compared to normal stroma in cancers including breast^14^, lung^15^ and ovarian^16–18^. In addition, genetic alterations in CAFs and tumour-associated stroma have been investigated in several cancer types, including ovarian cancer; however, these studies have concluded that somatic mutations are extremely rare^19–21^. This lack of somatic genetic alterations in the tumour stroma and CAFs raises the likelihood of alternative mechanisms of gene regulation. For example, differences in methylation between cancer-associated and normal stroma have been reported in breast, gastric and prostate cancer^22–25^. In addition, one study in ovarian cancer demonstrated that microRNAs differentially expressed in ovarian CAFs compared to normal omental fibroblasts can also regulate gene expression and play a role in the conversion of normal fibroblasts to CAFs^26^.

Long non-coding RNAs (lncRNAs) represent another potential candidate for regulating gene expression and function in CAFs that has yet to be explored. Once thought to be “transcriptional noise”, lncRNAs are non-protein coding RNAs >200 nucleotides in length that have relatively recently been shown to play vital roles in a large variety of biological functions such as genetic imprinting, chromatin modification and modulation of gene expression^27^. Increasingly, lncRNAs are being implicated in a variety of diseases, including cancer^28^. However, it has not been determined whether lncRNAs contribute to the phenotype and function of CAFs in any cancer. Given that the functions of many lncRNAs are still unknown, integrative computational approaches that discern different avenues of lncRNA functionality are highly desired^29^. Therefore, in this study we first sought to identify whether lncRNAs are differentially expressed in ovarian CAFs *versus* NOFs. We then developed a novel integrative network-based computational approach to identify those CAF-specific lncRNAs that may play a role in metastasis.

## Results

### Identification of lncRNAs differentially regulated in CAFs compared to NOFs

**Figure 1:**
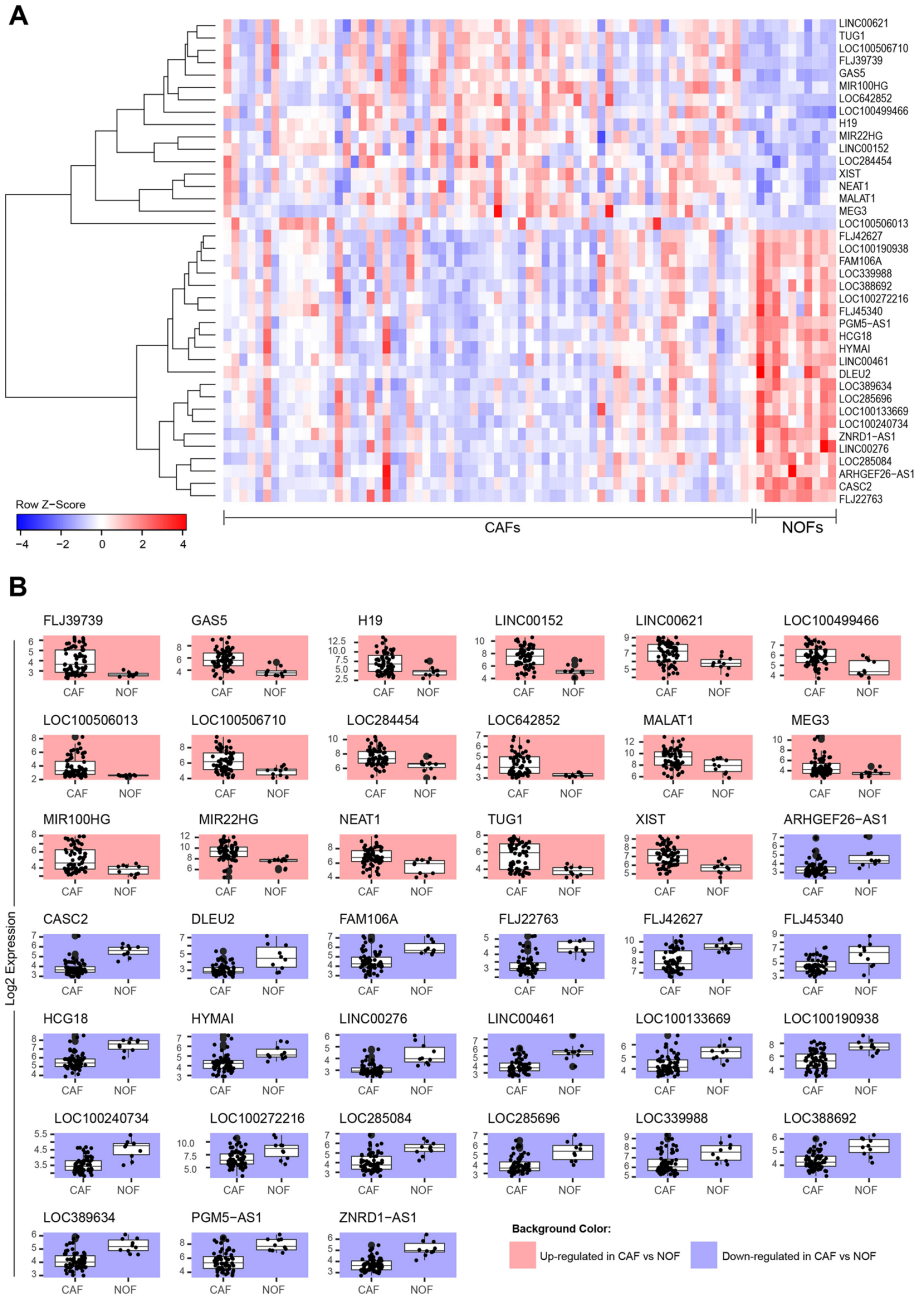
Differentially expressed lncRNAs in ovarian CAFs *vesus* NOFs. (**A**) Heatmap illustrating the expression profile differences of the identified lncRNAs differentially expressed in CAFs compared to NOFs. (**B**) Box plots showing the distribution of expression of each lncRNA in CAF samples compared to NOF samples.

To identify lncRNAs that were differentially expressed in CAFs versus NOFs, we analysed gene expression data from microdissected CAF samples obtained from 67 HGSOC patients and compared them to NOFs obtained from 10 patients who underwent oophorectomy for benign conditions. A significance cutoff of |log2(fold-change)| > 1 and p-value < 0.05 (adjusted p-value < 0.1) were used to identify lncRNAs differentially expressed in CAFs *versus* NOFs. In total, 60 probes were identified reflecting 39 unique lncRNAs with known titles (Table 1). A heatmap depicting the expression profiles of the identified lncRNAs in CAFs and NOFs is shown in Fig. 1A. Figure 1B depicts box plots representing the distributions of expressions of each lncRNA across CAF and NOF samples based on multiple statistics, *i.e*., minimum, median, maximum, and the interquartile range. Overall, 17 lncRNAs were up-regulated in CAFs and 22 lncRNAs were downregulated with evidently distinct distributions as the box plots display. Of the 39 lncRNAs differentially expressed in CAFs compared to NOFs, 11 were also differentially expressed in the corresponding laser-capture microdissected tumor epithelium compared to normal ovarian surface epithelium (Supplementary Table S1).

**Table 1:** lncRNAs differentially expressed in CAFs versus NOFs and the associated statistics. Positive fold change indicates upregulation in CAFs. lncRNA titles are extracted from Affymatrix U133 annotation file.

Symbol	LogFC	P-value	Adj p-val	Title
ARHGEF26-AS1	−1.209	1.82E-05	5.22E-04	ARHGEF26 antisense RNA 1 (non-protein coding)
CASC2	−1.751	3.74E-12	1.86E-08	cancer susceptibility candidate 2 (non-protein coding)
DLEU2	−1.539	1.51E-07	2.30E-05	deleted in lymphocytic leukemia 2 (non-protein coding)
FAM106A	−1.446	2.00E-05	5.54E-04	family with sequence similarity 106, member A
FLJ39739	1.297	1.12E-03	7.90E-03	uncharacterized FLJ39739
FLJ22763	−1.142	5.47E-08	1.16E-05	uncharacterized LOC401081
FLJ42627	−1.382	1.04E-04	1.63E-03	uncharacterized LOC645644
FLJ45340	−1.613	2.51E-05	6.43E-04	uncharacterized LOC402483
GAS5	2.09	2.82E-05	6.96E-04	growth arrest-specific 5 (non-protein coding)
H19	2.377	5.95E-03	2.46E-02	H19, imprinted maternally expressed transcript (non-protein coding)
HCG18	−1.826	1.44E-08	4.58E-06	HLA complex group 18 (non-protein coding)
HYMAI	−1.12	8.71E-05	1.46E-03	hydatidiform mole associated and imprinted (non-protein coding)
LINC00152	2.161	8.75E-05	1.46E-03	long intergenic non-protein coding RNA 152
LINC00276	−1.284	2.20E-10	2.93E-07	long intergenic non-protein coding RNA 276
LINC00461	−1.7	9.98E-08	1.75E-05	long intergenic non-protein coding RNA 461
LINC00621	1.213	5.81E-03	2.41E-02	long intergenic non-protein coding RNA 621
LOC100133669	−1.028	4.89E-05	1.00E-03	uncharacterized LOC100133669
LOC100190938	−2.25	7.88E-06	2.96E-04	uncharacterized LOC100190938
LOC100240734	−1.019	5.13E-08	1.13E-05	uncharacterized LOC100240734
LOC100272216	−1.803	9.46E-04	7.07E-03	uncharacterized LOC100272216
LOC100499466	1.22	1.71E-04	2.28E-03	uncharacterized LOC100499466
LOC100506013	1.226	7.05E-03	2.76E-02	uncharacterized LOC100506013
LOC100506710	1.367	2.21E-03	1.25E-02	endogenous Bornavirus-like nucleoprotein 2 pseudogene
LOC284454	1.16	2.00E-03	1.17E-02	uncharacterized LOC284454
LOC285084	−1.456	3.56E-06	1.78E-04	uncharacterized LOC285084
LOC285696	−1.413	2.94E-06	1.56E-04	uncharacterized LOC285696
LOC339988	−1.107	2.57E-03	1.39E-02	uncharacterized LOC339988
LOC388692	−1.048	1.87E-06	1.14E-04	uncharacterized LOC388692
LOC389634	−1.134	5.54E-07	5.04E-05	uncharacterized LOC389634
LOC642852	1.011	1.69E-03	1.05E-02	uncharacterized LOC642852
MALAT1	1.244	2.83E-02	7.40E-02	metastasis associated lung adenocarcinoma transcript 1 (non-protein coding)
MEG3	1.09	3.66E-02	8.93E-02	maternally expressed 3 (non-protein coding)
MIR100HG	1.347	5.28E-03	2.26E-02	mir-100-let-7a-2 cluster host gene (non-protein coding)
MIR22HG	1.846	2.38E-04	2.85E-03	MIR22 host gene (non-protein coding)
NEAT1	1.297	4.94E-03	1.88E-02	nuclear paraspeckle assembly transcript 1 (non-protein coding)
PGM5-AS1	−2.305	1.28E-06	8.91E-05	PGM5 antisense RNA 1 (non-protein coding)
TUG1	1.801	3.95E-04	3.98E-03	taurine upregulated 1 (non-protein coding)
XIST	1.439	1.19E-03	7.32E-03	X (inactive)-specific transcript (non-protein coding)
ZNRD1-AS1	−1.415	2.84E-09	1.48E-06	ZNRD1 antisense RNA 1 (non-protein coding)

### Predictive power of the differentially expressed lncRNAs to distinguish between CAFs and NOFs

**Figure 2:**
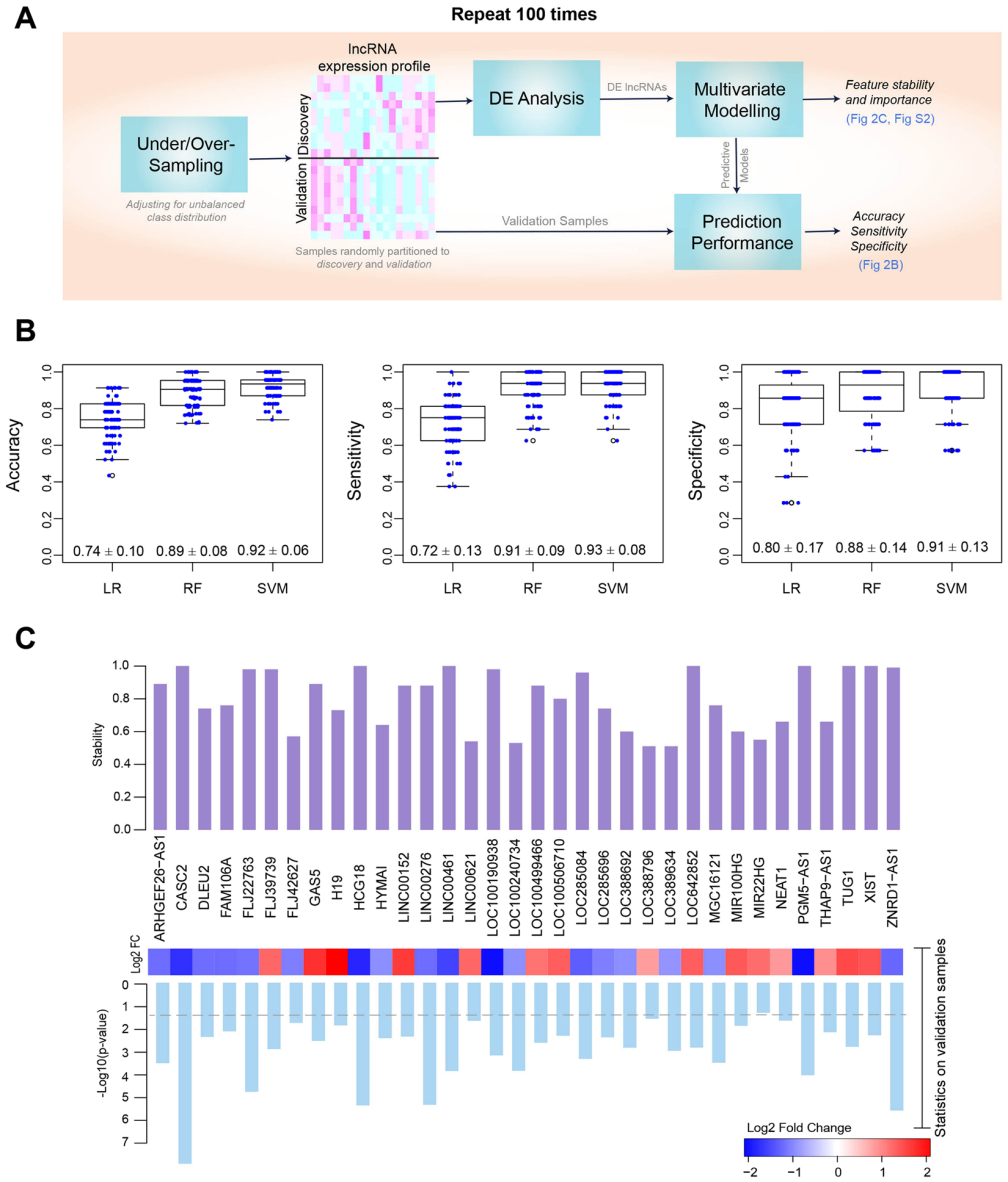
Predictive power of the identified lncRNAs. (**A**) Workflow used to assess the predictive power of differentially expressed lncRNAs (**B**) Box plots showing the distribution of accuracy, sensitivity and specificity of logistic regression (LR), random forest (RF), and Support Vector Machine (SVM) multivariate classification models across 100 iterations; for each box plot, the exact values of mean ± standard deviation are displayed (**C**) Bar plots representing the ‘feature stability’ of 34 lncRNAs identified to be differentially expressed in 50 iterations or more. The feature stability is defined as the proportion of runs that a lncRNA is identified to be differentially expressed and thus selected as a feature for the multivariate predictive models. More stable features are less sensitive to data partitioning. Log fold change and p-values of lncRNAs in validation sets were estimated and averaged across 100 iterations. Dashed line on p-value bar chart is the indicator of the 0.05 cutoff.

To accurately assess the predictive power of the differentially expressed lncRNAs to distinguish between CAFs and NOFs, the unbalanced class distribution was first adjusted using SMOTE algorithm^30^, a well-known oversampling machine learning technique. We oversampled NOFs, increased sample size by 50%, and undersampled CAFs, decreased samples by 50% which resulted in 15 NOFs and 33 CAFs for classification analyses. Supplementary Fig. S1 visualises the original and oversampled/undersampled dataset by plotting the samples across the first two principle components of DE lncRNAs. Highlighted NOFs are those generated using SMOTE oversampling. Samples were then randomly partitioned into discovery and validation sets and were analysed according to the workflow outlined in Fig. 2A. Logistic regression (LR), Random Forest (RF) and Support Vector Machine (SVM) multivariate models whose predictors are differentially expressed lncRNAs across the discovery samples were trained on the discovery set. The quality of the models in distinguishing CAFs and NOFs was then assessed by estimating accuracy, sensitivity, and specificity measures on the validation set. Boxplots in Fig. 2B show the distributions of these measures over 100 iterations of randomly partitioning samples to discovery and validation sets. The SVM model significantly outperformed the LR model in all measures and the RF model in accuracy and sensitivity measures (Wilcoxon *p-*value < 10E-5). The average specificity of SVM is 0.91 ± 0.13 confirming the ability and stability of this model in predicting underrepresented NOF samples. It also demonstrated the average accuracy of 0.92 ± 0.06 confirming the overall discriminatory power of the identified lncRNAs.

Differentially expressed lncRNAs are subject to change across iterations due to the change in the composition of the discovery set. We chose *stable* lncRNAs as those identified to be differentially expressed in more than 50% of iterations. Accordingly, 34 lncRNAs were selected, including all lncRNAs listed in Table 1 except for *FLJ45340*, *LOC100272216*, *LOC100506013*, *LOC284454*, *LOC339988*, *MALAT1*, and *MEG3* which identified as differentially expressed in 38, 29, 38, 3, 26, 20, and 26 iterations, respectively. For each of these 34 lncRNAs, Fig. 2C plots the feature “stability” as the proportion of runs where the corresponding lncRNA was differentially expressed and thus selected as a feature of the predictive models. Figure 2C also shows the fold change values and p-values of these lncRNAs comparing CAFs versus NOFs in the validation sets averaged across iterations. The contribution of each lncRNA in the prediction performance of the RF model was estimated across iterations and visualised in Supplementary Fig. S2, which shows the overall positive contribution of lncRNAs in the model accuracy. Additionally, the predictive power of each of 38 lncRNAs was individually assessed using a univariate LR model whose sole predictor is the lncRNA expression across discovery samples (Table S2). The results highlight the advantage of using multivariate predictive models and confirm the previous findings that compared to single molecules, molecular signatures are more robust biomarkers and more powerful predictors of disease phenotypes^31^.

### Functional roles of the identified lncRNAs in metastasis

**Figure 3:**
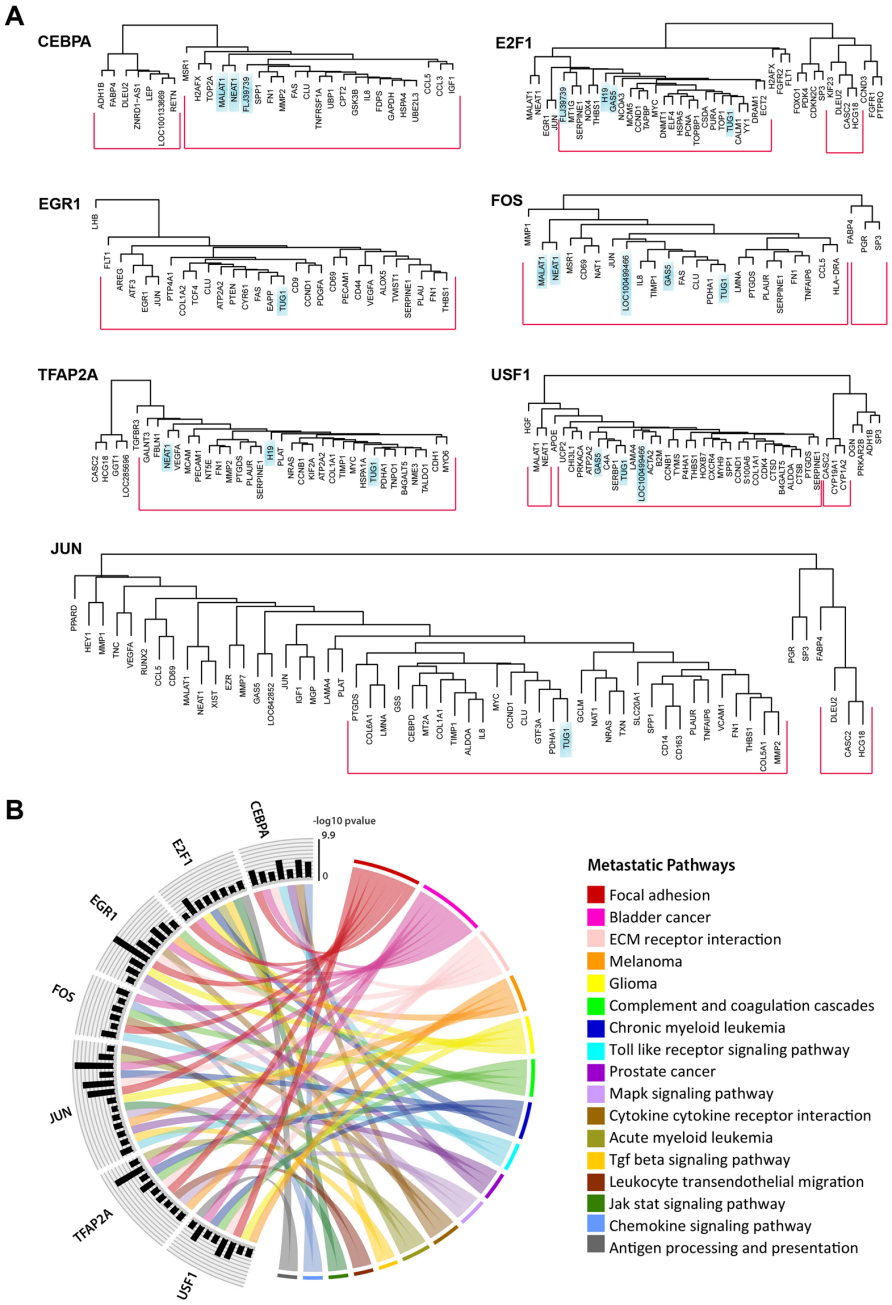
Functional prediction of CAF-associated lncRNAs. (**A**) Hierarchical clustering dendrograms showing the lncRNAs in the predominant co-regulatory module for each TF. (**B**) Metastatic pathways enriched by TFs’ predominant clusters; links between a TF and a pathway represents the enrichment of the pathway by targets of the TF in the prevalent cluster; histogram bars show the corresponding p-value log10 scaled and negated to improve readability.

The functional roles of deregulated lncRNAs in ovarian CAFs were predicted by first constructing the context-specific regulatory network of transcription factor (TF)-lncRNA and TF-target gene (TG) interactions. The network comprises 42 enriched TFs targeting 31 lncRNAs and 646 TGs differentially expressed in CAFs *versus* NOFs, totaling 1,266 interactions (375 TF-lncRNA and 891 TF-TG interactions). The network flat file is available in Supplementary Data and visualised in Supplementary Fig. S1. For each TF, targets with a similar expression pattern across all samples clustered together (using hierarchical clustering), forming coregulatory modules (clusters with p-value < 0.05). All targets within the same module are regulated by the same TF and expressed consistently across samples and thus assumed to function correspondingly. The focus of this study is on the metastatic role of lncRNAs. Pathways expressed in ovarian cancer metastases have been identified previously^32^; all metastasis-associated pathways considered in this study have been provided in Supplementary Table S3. An enrichment analysis was performed to identify metastasis-related pathways overrepresented by TGs within each module. Figure 3 shows TFs whose predominant coregulatory module (*i.e*., the largest cluster) enriches multiple (>5) metastatic pathways and comprises at least one lncRNA yet the number of lncRNAs is less than the number of coregulated TGs. Figure 3 demonstrates the association of 7 lncRNAs upregulated in CAFs (*FLJ39739*, *GAS5*, *H19*, *LOC100499466*, *MALAT1*, *NEAT1*, and *TUG1*) with multiple pathways in ovarian cancer metastasis including pathways involved in the interaction of cells with the microenvironment, namely *focal adhesion*, and *ECM receptor interaction*. We also identified co-regulatory modules containing lncRNAs whose downregulation was associated with enrichment of metastasis-associated pathways. These included lncRNAs *CASC2, DLEU2, HCG18* and *LOC100133669*. Complete results including clusters formed by all TFs’ targets along with the corresponding enriched pathways are available in Supplementary Table S4.

## Discussion and Conclusion

The tumour microenvironment and more specifically CAFs are now accepted to play an important role in several aspects of tumour development and progression in many cancer types, including ovarian. The relative genetic stability of CAFs also makes them an attractive therapeutic target, but given this genetic stability, the factors that regulate gene expression in ovarian CAFs are not well elucidated. We sought to identify whether lncRNAs are involved in the CAF phenotype, firstly by comparing lncRNA expression profiles of ovarian CAFs to NOFs in order to identify those lncRNAs that could be used to confidently differentiate CAFs from NOFs. Then, given the known role of CAFs in promoting metastasis, we hypothesised that some of the differentially expressed lncRNAs may play a role in the pro-metastatic phenotype of CAFs. As the function of many lncRNAs is still not well known, we took an integrative computational approach to identify those lncRNAs which may be involved in the pro-metastatic function of CAFs.

To date, studies analysing lncRNA expression in ovarian cancer have identified a number that are aberrantly expressed^33–35^. However, these studies used ovarian cancer samples from bulk tumour; therefore the involvement of lncRNAs specifically in tumour stroma or CAFs could not be determined. By using gene expression data from laser-capture microdissected samples, we were able to identify 39 lncRNAs aberrantly expressed in ovarian CAFs compared to NOFs. This is likely an underestimate of the total number of lncRNAs that are differentially expressed between CAFs and NOFs due to the limited number of probes corresponding to lncRNAs on the array used. Of the lncRNAs identified, there were several that have previously been implicated in ovarian cancer. These include *NEAT1* ^36, 37^, *TUG1* ^38^, *MALAT1* ^39^, *H19* ^40–42^, *XIST* ^43, 44^, *GAS5* ^45, 46^ and *MEG3* ^47^. Also included were lncRNAs reported to play roles in other cancer types but that have not previously been associated with ovarian cancer such as *FLJ39739* ^48^, *DLEU2* ^49^, *CASC2* ^50, 51^, *LINC00152* ^52^, *MIR100HG* ^53, 54^ and *MIR22HG* ^55^. Our data suggest that these lncRNAs could play a role in ovarian CAFs rather than, or as well as, the tumour cells themselves. This is supported by the fact that 28 out of the 39 differentially expressed lncRNAs were not differentially expressed in tumour epithelium compared to ovarian surface epithelium. This is the first report in ovarian cancer to show lncRNAs to be aberrantly expressed in the tumour stroma. This is also likely to be the case in other tumour types; however, to date this has not been widely investigated. A recent study by Zhang *et al*. used chromogenic *in situ* hybridisation to examine a panel of 6 lncRNAs previously implicated in breast cancer in order to identify the cellular components expressing each lncRNA^56^. Interestingly, the lncRNAs *H19* and *MEG3* were shown to be overexpressed in the breast tumour stroma rather than the tumour cells, while *MALAT1* was found to be expressed in both tumour and stromal tissue. While limited to a panel of 6 lncRNAs, these data strongly support our findings in ovarian CAFs and suggest that ovarian CAF-associated lncRNAs may also play a role in CAFs from other tumour types. Furthermore, CAFs are thought to most strongly resemble activated myofibroblasts, which are also increased in other pathologies such as liver fibrosis. Two lncRNAs identified in our study as differentially expressed in CAFs have previously been shown to be increased in activated hepatic stellate cells: *MALAT1* ^57, 58^ and *GAS5* ^59^.

The identification of several lncRNAs differentially expressed in ovarian CAFs supports our hypothesis that lncRNAs play a role in regulating gene expression in CAFs. We then wished to determine whether any of these lncRNAs may play a role in the pro-tumorigenic function of CAFs. CAFs have been shown to influence the behavior of tumour cells in several ways, a major role of which is to promote metastasis. We therefore constructed a context specific interaction network to identify those lncRNAs that play a role in the pro-metastatic phenotype of CAFs. This led to the identification of several metastasis-associated lncRNAs, several of which have been implicated in cancer metastasis but have not previously been associated with the metastatic role of CAFs. In addition, these lncRNAs clustered with several genes that have been shown to be overexpressed in CAFs and contribute to their ability to promote metastasis such as *MMP2* ^60^ *and IL8* ^61^. In ovarian cancer, overexpression of *NEAT1* ^36, 37^, *TUG1* ^38^ and *MALAT1* ^39^ have previously been associated with increased tumour grade, FIGO stage and an increase in distant metastasis. However, these studies used whole tumour specimens for analysis; therefore, the role of these lncRNAs in CAFs was not determined. Interestingly, these three lncRNAs have all been shown to regulate epithelial to mesenchymal transition in multiple cancer types *in vitro* ^62–65^, which is a process necessary for cancer cells to metastasise but is also known to be regulated by CAFs. It is possible that the ability for CAFs to promote epithelial to mesenchymal transition in tumour cells could in part be mediated by these lncRNAs and warrants further investigation.

CAFs are well known to play important roles in cancer and metastasis and as such, represent an attractive target for novel therapies in multiple cancer types. However, a better understanding of the molecular factors that differentiate CAFs from normal fibroblasts is essential for the development of therapies that specifically target CAFs. While gene expression profiling studies have determined that CAFs differ from normal fibroblasts, the lack of genetic mutations suggests that other complex mechanisms of gene regulation are at play in the tumour microenvironment. For the first time in any cancer, we have shown that lncRNAs represent one possible mechanism of gene expression regulation in CAFs that can be used to differentiate them from NOFs. Furthermore, several of these CAFs are also predicted play a role in the metastasis-promoting phenotype of CAFs. These data provide a greater understanding of the complexities involved in the CAF phenotype, despite their genetic stability and may lead to the design of targeted CAF therapies.

## Materials and Methods

### Tissue Specimens

Primary tumour specimens from 67 women diagnosed with HGSOC were obtained as previously described^18, 66^. All specimens were from HGSOC patients prior to treatment hospitalised at the Brigham and Women’s Hospital between 1990 and 2000. All specimens and their corresponding clinical information were collected by written consent under protocols approved by the review board of the Brigham and Women’s Hospital Ethics Committee. All procedures were performed in accordance with the approved guidelines and regulations. Classification was determined according to the International Federation of Gynecology and Obstetrics (FIGO) standards. Normal ovaries were obtained from 10 patients who underwent surgery for benign gynaecologic conditions. The characteristics of tumour samples included in this study are shown in Table 2. There was no significant difference between the ages of the oophorectomy patients (mean age ± Std = 62.3 ± 5.272) and the HGSOC patients at time of surgery (P = 0.674, Mann Whitney U Test).

**Table 2:** Clinical characteristics of tumour samples.

Characteristics	n = 67
Age at diagnosis (mean ± std)	60.98 ± 12.28 years
Stage (III/IV), Grade	55/8, 3
Site & Histological types	Ovary, Serous
Chemoresponse (R/S/R-S/Ref)*	18/24/7/4

*R: Resistant, S: Sensitive, R-S: Resistant-Sensitive, R: Refractory.

### Microdissection, RNA isolation, amplification and hybridisation

Microdissection, RNA isolation, amplification and hybridisation to GeneChip Human Genome U133 Plus 2.0 Oligonucleotide arrays (Affymetrix) were performed as described previously^18^. Gene expression of endothelial cell markers (*TIE-2* and *VEGFR1*) and T cell markers (*CD8* and *CD45*) were below the level of detection in the samples, indicating a lack of immune or endothelial components of the stroma and enrichment for fibroblasts^18^. All gene array data are available through Gene Expression Omnibus (GEO) accession number GSE40595.

### Data pre-processing and differential expression analysis

Data pre-processing was performed using R Bioconducter,’affy’ package. Data were normalised and background corrected using the Robust Multi-Array Average method^67^ and expression values Log2 transformed. Differentially expressed probes (both lncRNAs and protein-coding genes) between CAFs *versus* NOFs were identified using the moderated t-test as defined in the Bioconductor *limma* package^68^; *p*-values were adjusted for multiple hypothesis testing using the False Discovery Rate (FDR) correction. The 2,448 probes corresponding to 1,970 lncRNAs were identified previously by Zhang *et al*.^69^. The gene symbols and titles corresponding to these probes were matched by Affymetrix U133 Plus 2.0 Array annotation file.

### Analysis of the predictive power of the identified lncRNAs

A well-balanced dataset is critical for creating powerful prediction models as most existing classifiers tends to optimise the overall prediction accuracy and thus perform poorly on the minority class examples when the dataset is very imbalanced. We adjusted for the imbalanced class distribution between CAFs and NOFs using an oversampling/undersampling machine learning approach typically used to construct classifiers from unbalanced datasets^70^. Over/undersampling techniques have shown improved prediction performance when used to handle imbalanced clinical datasets^71, 72^. We used *SMOTE*: *Synthetic Minority Oversampling Technique* ^30^, a well-known and powerful technique which synthetically generates new examples of the minority class using the nearest neighbours of the cases and randomly samples from the majority class examples in order to produce a more balanced dataset. SMOTE was implemented using R package ‘DMwR’.

Once the original data was over/undersampled to produce a more balanced dataset, to assess the predictive power of differentially expressed lncRNAs, samples were first randomly partitioned into two disjoint sets of *discovery* (50% of samples) and *validation* (50% of samples). LncRNAs differentially expressed in the discovery set (i.e., |log2(fold-change)| > 1 and p-value < 0.05) were then selected as features/predictors of three widely-used multivariate predictive models namely *logistic regression* (LR)^73^, *Random Forest* (RF)^74^, and *Support Vector Machine* (SVM)^75^ with linear kernel function. All models were trained on the discovery set. The resultant models were then used to predict CAFs *versus* NOFs based on the expression values of identified lncRNAs in validation samples. The quality of the models was assessed based on the standard statistical performance measures of classification tests, *i.e*., accuracy, sensitivity and specificity. The accuracy is the proportion of true predictions (both CAFs and NOFs) among the total number of cases in the validation set. Sensitivity measures the proportion of CAFs in the validation set that are correctly identified as such. Specificity measures the proportion of NOFs in the validation set that are correctly identified as such. For statistical rigour, to account for random partitioning of the samples into discovery and validation sets, the whole process was repeated 100 times and the averaged measures of performance were reported. The importance or relative contribution of each feature (differentially expressed lncRNA) in the RF performance was estimated based on the ‘*mean decrease accuracy*’ measure^74^ as described in Supplementary Fig. S2. All analyses were performed using R ‘caret’, ‘RandomForest, and ‘e1071’packages.

### Network-based functional analysis of the identified lncRNAs

#### Construction of a context-specific network of transcription factor (TF)-lncRNA and TF-gene regulations

TF-lncRNA regulatory relationships were extracted from ChIPBase database, which provides a comprehensive annotation of TF-lncRNA interaction map using ChIP-Seq data generated from multiple organisms^76^. We searched ChIPBase using default parameters (*i.e*., regulatory region of 5 kb upstream and 1 kb downstream) and filtered for mammalian organisms to obtain the list of TF interacting with the identified lncRNAs. The names of the interacting TFs were disambiguated using NCBI to obtain their official symbols provided by the HGNC (HUGO Gene Nomenclature Committee). These TFs were used to search ORTI^77^, a recently compiled repository of mammalian transcriptional interactions, to obtain their experimentally-validated (Rank 1) target genes (TGs). The compiled TF-TG interactions are sourced from a range of experimental conditions. In order to identify TFs modulated under the specific context of the study, we used ORTI application 1^77^, which performs TF enrichment analysis on a list of genes differentially expressed in CAFs *versus* NOFs (*p*-value < 0.05 and |log2(fold-change)| > 1, using a similar moderated t-test employed to derive differentially expressed lncRNAs). TFs with enrichment *p-*values < 0.05 were then considered as ‘active’ in the context of the study. Subsequently, a context-specific regulatory network consisting of interactions between enriched TFs and differentially expressed TGs and lncRNAs was constructed for the subsequent analysis.

#### Prediction of lncRNA functions

The functional roles of lncRNAs deregulated in ovarian CAFs were characterised based on the functions of the co-expressed TGs using the constructed context-specific regulatory network. Accordingly, for each TF, targets with highly correlated expression patterns were clustered together and assumed to conduct similar functions (in the context of study) given that they were targeted by an identical ‘active’ TF and followed similar expression pattern across different samples. We performed hierarchical clustering on expression profiles of targets of each TF using R ‘pvclust’ package which conducts multiscale bootstrap resampling to calculate *p*-values for each cluster. Sample correlation was chosen as the distance measure and the ‘average’ agglomerative method was used in the hierarchical clustering. Clusters whose estimated p*-*value was < 0.05 were identified as co-regulatory modules of the TF.

**Figure 4:**
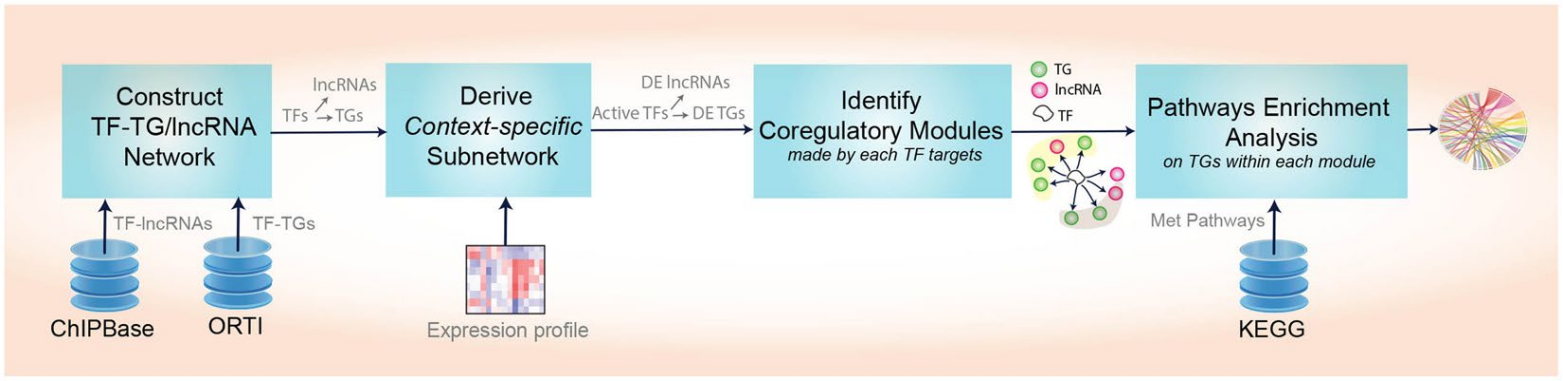
Workflow for prediction of lncRNA function. Schematic diagram depicting the steps involved in construction of the context-specific network and downstream functional analysis.

The functional roles of lncRNAs were then predicted using the functions of the co-regulating TGs. In this study, we were focused on the metastatic role of lncRNAs in ovarian CAFs. Pathways expressed in metastatic ovarian tumours were identified previously^32^. We chose KEGG pathways whose enrichment p*-*value was <0.05 and performed pathway enrichment analysis on genes involved in each co-regulatory module using the right-sided Fisher’s exact test where the p-value for the null hypothesis is computed based on the hypergeometric distribution. Consequently, lncRNAs in each module were predicted to be involved in the metastatic pathways enriched (adjusted p-value < 0.1) by the co-regulating TGs in the module. The entire workflow for the network and subsequent functional analysis is depicted in Fig. 4.

## Electronic supplementary material

Supplementary Information

Dataset 1
