# Supplementary material for: Functional prediction of long non-coding RNAs in ovarian cancer-associated fibroblasts indicate a potential role in metastasis
Source: Sci Rep. 2017 Sep 4;7:10374. doi: 10.1038/s41598-017-10869-y (PMC5583324; doi:10.1038/s41598-017-10869-y)

Supplementary Information

Functional prediction of long non-coding RNAs in ovarian cancer-associated fibroblasts indicate a potential role in metastasis

Fatemeh Vafaee[1]*, Emily K. Colvin[2,3]*[§], Samuel C. Mok[4], Viive M. Howell[2,3], Goli Samimi[5]

[1]School of Biotechnology and Biomolecular Sciences, University of New South Wales, NSW 2052, Australia

[2]Bill Walsh Translational Cancer Research Laboratory, Kolling Institute, Northern Sydney Local Health District, St Leonards NSW 2065, Australia

[3]Sydney Medical School Northern, University of Sydney, NSW 2006, Australia

[4]Department of Gynecologic Oncology and Reproductive Medicine Research, Division of Surgery, The University of Texas MD Anderson Cancer Center, Houston, TX

[5]Division of Cancer Prevention, National Cancer Institute, National Institutes of Health, Bethesda, MD

*These authors contributed equally to this work

[§]Corresponding author

emily.colvin@sydney.edu.au

Ph: +61 2 9926 4846

Supplementary Table S1:

lncRNAs deregulated in CAFs vs NOFs and Tumor: Of the 39 lncRNAs differentially expressed in CAFs compared to NOFs, 11 (highlighted) were also differentially expressed in matched laser-capture microdissected tumor epithelium compared to normal ovarian surface epithelium.

	CAF vs NOF			Tumor vs Normal			
**Symbol**	**LogFC**	**P-value**	**Adj p-val**	**LogFC**	**P-value**	**Adj p-val**	**Title**
**ARHGEF26-AS1**	-1.209	1.82E-05	5.22E-04	-0.234	1.90E-01	2.58E-01	ARHGEF26 antisense RNA 1 (non-protein coding)
**CASC2**	-1.751	3.74E-12	1.86E-08	0.148	1.91E-02	3.80E-02	cancer susceptibility candidate 2 (non-protein coding)
**DLEU2**	-1.539	1.51E-07	2.30E-05	1.99	6.66E-14	3.89E-12	deleted in lymphocytic leukemia 2 (non-protein coding)
**FAM106A**	-1.446	2.00E-05	5.54E-04	0.0987	5.40E-01	6.14E-01	family with sequence similarity 106, member A
**FLJ22763**	-1.142	5.47E-08	1.16E-05	0.512	9.27E-02	1.41E-01	uncharacterized LOC401081
**FLJ39739**	1.297	1.12E-03	7.90E-03	-0.233	6.20E-04	2.39E-03	uncharacterized FLJ39739
**FLJ42627**	-1.382	1.04E-04	1.63E-03	0.471	2.82E-03	8.04E-03	uncharacterized LOC645644
**FLJ45340**	-1.613	2.51E-05	6.43E-04	0.553	7.47E-04	2.78E-03	uncharacterized LOC402483
**GAS5**	2.09	2.82E-05	6.96E-04	0.467	1.19E-01	1.74E-01	growth arrest-specific 5 (non-protein coding)
**H19**	2.377	5.95E-03	2.46E-02	-0.588	5.30E-01	6.04E-01	H19, imprinted maternally expressed transcript (non-protein coding)
**HCG18**	-1.826	1.44E-08	4.58E-06	-0.0349	7.99E-01	8.39E-01	HLA complex group 18 (non-protein coding)
**HYMAI**	-1.12	8.71E-05	1.46E-03	1.49	6.35E-13	3.00E-11	hydatidiform mole associated and imprinted (non-protein coding)
**LINC00152**	2.161	8.75E-05	1.46E-03	-0.0061	9.82E-01	9.86E-01	long intergenic non-protein coding RNA 152
**LINC00276**	-1.284	2.20E-10	2.93E-07	-0.0285	6.82E-01	7.40E-01	long intergenic non-protein coding RNA 276
**LINC00461**	-1.7	9.98E-08	1.75E-05	-0.186	1.37E-02	2.89E-02	long intergenic non-protein coding RNA 461
**LINC00621**	1.213	5.81E-03	2.41E-02	-0.354	3.09E-01	3.87E-01	long intergenic non-protein coding RNA 621
**LOC100133669**	-1.028	4.89E-05	1.00E-03	-0.153	1.20E-01	1.75E-01	uncharacterized LOC100133669
**LOC100190938**	-2.25	7.88E-06	2.96E-04	0.401	4.37E-03	1.14E-02	uncharacterized LOC100190938
**LOC100240734**	-1.019	5.13E-08	1.13E-05	-0.108	3.16E-01	3.94E-01	uncharacterized LOC100240734
**LOC100272216**	-1.803	9.46E-04	7.07E-03	2.08	2.23E-10	5.84E-09	uncharacterized LOC100272216
**LOC100499466**	1.22	1.71E-04	2.28E-03	1.12	8.65E-05	4.85E-04	uncharacterized LOC100499466
**LOC100506013**	1.226	7.05E-03	2.76E-02	-0.684	2.65E-02	4.98E-02	uncharacterized LOC100506013
**LOC100506710**	1.367	2.21E-03	1.25E-02	1.08	4.71E-04	1.91E-03	endogenous Bornavirus-like nucleoprotein 2 pseudogene
**LOC284454**	1.16	2.00E-03	1.17E-02	-0.626	8.07E-03	1.88E-02	uncharacterized LOC284454
**LOC285084**	-1.456	3.56E-06	1.78E-04	-0.404	7.41E-05	4.28E-04	uncharacterized LOC285084
**LOC285696**	-1.413	2.94E-06	1.56E-04	-0.27	1.16E-02	2.52E-02	uncharacterized LOC285696
**LOC339988**	-1.107	2.57E-03	1.39E-02	0.0781	4.66E-01	5.43E-01	uncharacterized LOC339988
**LOC388692**	-1.048	1.87E-06	1.14E-04	0.94	1.86E-06	1.83E-05	uncharacterized LOC388692
**LOC389634**	-1.134	5.54E-07	5.04E-05	-0.17	7.62E-02	1.20E-01	uncharacterized LOC389634
**LOC642852**	1.011	1.69E-03	1.05E-02	0.778	6.64E-03	1.60E-02	uncharacterized LOC642852
**MALAT1**	1.244	2.83E-02	7.40E-02	0.782	9.97E-02	1.49E-01	metastasis associated lung adenocarcinoma transcript 1 (non-protein coding)
**MEG3**	1.09	3.66E-02	8.93E-02	2.39	8.34E-09	1.51E-07	maternally expressed 3 (non-protein coding)
**MIR100HG**	1.347	5.28E-03	2.26E-02	2.08	2.39E-05	1.65E-04	mir-100-let-7a-2 cluster host gene (non-protein coding)
**MIR22HG**	1.846	2.38E-04	2.85E-03	2.51	6.15E-11	1.85E-09	MIR22 host gene (non-protein coding)
**NEAT1**	1.297	4.94E-03	1.88E-02	1.42	6.11E-04	2.36E-03	nuclear paraspeckle assembly transcript 1 (non-protein coding)
**PGM5-AS1**	-2.305	1.28E-06	8.91E-05	0.895	3.43E-07	4.14E-06	PGM5 antisense RNA 1 (non-protein coding)
**TUG1**	1.801	3.95E-04	3.98E-03	1.07	3.51E-04	1.51E-03	taurine upregulated 1 (non-protein coding)
**XIST**	1.439	1.19E-03	7.32E-03	2.84	8.08E-05	4.04E-04	X (inactive)-specific transcript (non-protein coding)
**ZNRD1-AS1**	-1.415	2.84E-09	1.48E-06	0.328	9.81E-03	2.20E-02	ZNRD1 antisense RNA 1 (non-protein coding)

Supplementary Table S2:

Prediction performance of individual lncRNAs using univariate logistic regression models. Format: mean ± standard deviation across 100 iterations of partitioning samples to discovery and validation sets.

Symbol	Accuracy	Sensitivity	Specificity
**ARHGEF26-AS1**	0.813 ± 0.051	0.884 ± 0.065	0.651 ± 0.213
**CASC2**	0.950 ± 0.038	0.974 ± 0.031	0.896 ± 0.123
**DLEU2**	0.773 ± 0.069	0.893 ± 0.094	0.497 ± 0.144
**FAM106A**	0.733 ± 0.048	0.864 ± 0.084	0.433 ± 0.211
**FLJ22763**	0.853 ± 0.057	0.878 ± 0.083	0.794 ± 0.176
**FLJ39739**	0.794 ± 0.064	0.793 ± 0.093	0.799 ± 0.149
**FLJ42627**	0.703 ± 0.055	0.82 ± 0.106	0.434 ± 0.218
**FLJ45340**	0.753 ± 0.055	0.888 ± 0.08	0.443 ± 0.161
**GAS5**	0.840 ± 0.054	0.886 ± 0.068	0.734 ± 0.167
**H19**	0.716 ± 0.061	0.810 ± 0.107	0.500 ± 0.218
**HCG18**	0.881 ± 0.046	0.921 ± 0.069	0.789 ± 0.148
**HYMAI**	0.856 ± 0.054	0.936 ± 0.087	0.673 ± 0.209
**LINC00152**	0.830 ± 0.062	0.883 ± 0.09	0.707 ± 0.126
**LINC00276**	0.891 ± 0.052	0.929 ± 0.069	0.804 ± 0.154
**LINC00461**	0.832 ± 0.059	0.861 ± 0.085	0.764 ± 0.141
**LINC00621**	0.727 ± 0.056	0.837 ± 0.085	0.474 ± 0.22
**LOC100133669**	0.769 ± 0.055	0.859 ± 0.083	0.563 ± 0.216
**LOC100190938**	0.776 ± 0.055	0.832 ± 0.086	0.649 ± 0.173
**LOC100240734**	0.839 ± 0.054	0.909 ± 0.062	0.680 ± 0.166
**LOC100272216**	0.695 ± 0.065	0.858 ± 0.118	0.323 ± 0.192
**LOC100499466**	0.833 ± 0.057	0.906 ± 0.07	0.666 ± 0.126
**LOC100506013**	0.807 ± 0.063	0.813 ± 0.104	0.791 ± 0.170
**LOC100506710**	0.746 ± 0.06	0.836 ± 0.098	0.539 ± 0.224
**LOC284454**	0.672 ± 0.054	0.918 ± 0.086	0.111 ± 0.094
**LOC285084**	0.792 ± 0.066	0.839 ± 0.107	0.684 ± 0.179
**LOC285696**	0.768 ± 0.059	0.868 ± 0.09	0.540 ± 0.185
**LOC339988**	0.706 ± 0.071	0.846 ± 0.124	0.386 ± 0.173
**LOC388692**	0.800 ± 0.071	0.867 ± 0.107	0.649 ± 0.150
**LOC389634**	0.799 ± 0.064	0.864 ± 0.095	0.649 ± 0.167
**LOC642852**	0.868 ± 0.052	0.904 ± 0.087	0.786 ± 0.123
**MALAT1**	0.694 ± 0.060	0.883 ± 0.105	0.261 ± 0.145
**MIR100HG**	0.680 ± 0.059	0.817 ± 0.118	0.367 ± 0.204
**MIR22HG**	0.708 ± 0.072	0.808 ± 0.105	0.479 ± 0.16
**NEAT1**	0.716 ± 0.055	0.859 ± 0.112	0.389 ± 0.331
**PGM5-AS1**	0.784 ± 0.059	0.833 ± 0.099	0.673 ± 0.195
**TUG1**	0.828 ± 0.051	0.870 ± 0.086	0.733 ± 0.187
**XIST**	0.713 ± 0.057	0.778 ± 0.096	0.566 ± 0.182
**ZNRD1-AS1**	0.871 ± 0.055	0.869 ± 0.088	0.876 ± 0.15

Supplementary Table S3:

Pathways deregulated in Metastasis.

KEGG Gene Sets up-regulated in Mets		
**Name**	**Size**	**FDR q-value**
ECM RECEPTOR INTERACTION	71	0
FOCAL ADHESION	163	0
CELL ADHESION MOLECULES CAMS	95	0
PRIMARY IMMUNODEFICIENCY	25	0
COMPLEMENT AND COAGULATION CASCADES	39	0
HEMATOPOIETIC CELL LINEAGE	58	0
CYTOKINE CYTOKINE RECEPTOR INTERACTION	162	0.00031
CALCIUM SIGNALING PATHWAY	130	0.000814
NATURAL KILLER CELL MEDIATED CYTOTOXICITY	93	0.001333
REGULATION OF ACTIN CYTOSKELETON	163	0.001546
AUTOIMMUNE THYROID DISEASE	24	0.001735
BLADDER CANCER	36	0.001892
FC GAMMA R MEDIATED PHAGOCYTOSIS	77	0.001927
LEUKOCYTE TRANSENDOTHELIAL MIGRATION	89	0.003847
MELANOMA	58	0.004314
INTESTINAL IMMUNE NETWORK FOR IGA PRODUCTION	32	0.005587
ANTIGEN PROCESSING AND PRESENTATION	53	0.00588
VIRAL MYOCARDITIS	52	0.006146
DILATED CARDIOMYOPATHY	75	0.013478
ALLOGRAFT REJECTION	23	0.016963
PPAR SIGNALING PATHWAY	52	0.017557
NEUROACTIVE LIGAND RECEPTOR INTERACTION	132	0.020473
B CELL RECEPTOR SIGNALING PATHWAY	64	0.020642
HYPERTROPHIC CARDIOMYOPATHY HCM	68	0.021176
GRAFT VERSUS HOST DISEASE	28	0.021272
MAPK SIGNALING PATHWAY	222	0.031174
ARRHYTHMOGENIC RIGHT VENTRICULAR CARDIOMYOPATHY ARVC	61	0.032618
CHEMOKINE SIGNALING PATHWAY	144	0.033308
ACUTE MYELOID LEUKEMIA	49	0.033486
ENDOCYTOSIS	134	0.033708
AXON GUIDANCE	109	0.034533
VASCULAR SMOOTH MUSCLE CONTRACTION	98	0.041691
TGF BETA SIGNALING PATHWAY	73	0.042688
LEISHMANIA INFECTION	49	0.042946
T CELL RECEPTOR SIGNALING PATHWAY	85	0.046791
**KEGG Gene Sets down-regulated in Mets**		
**Name**	**Size**	**FDR q-value**
RNA DEGRADATION	44	0.00782348
PROPANOATE METABOLISM	25	0.009993404
NITROGEN METABOLISM	18	0.018406615
VALINE LEUCINE AND ISOLEUCINE DEGRADATION	39	0.02016364
UBIQUITIN MEDIATED PROTEOLYSIS	101	0.023664232
TERPENOID BACKBONE BIOSYNTHESIS	11	0.044234212

Supplementary Figure S1:

PCA analysis on the original and oversampled/undersampled dataset: samples are plotted across the first two principle components of DE lncRNAs. Highlighted NOFs are those generated using SMOTE oversampling.

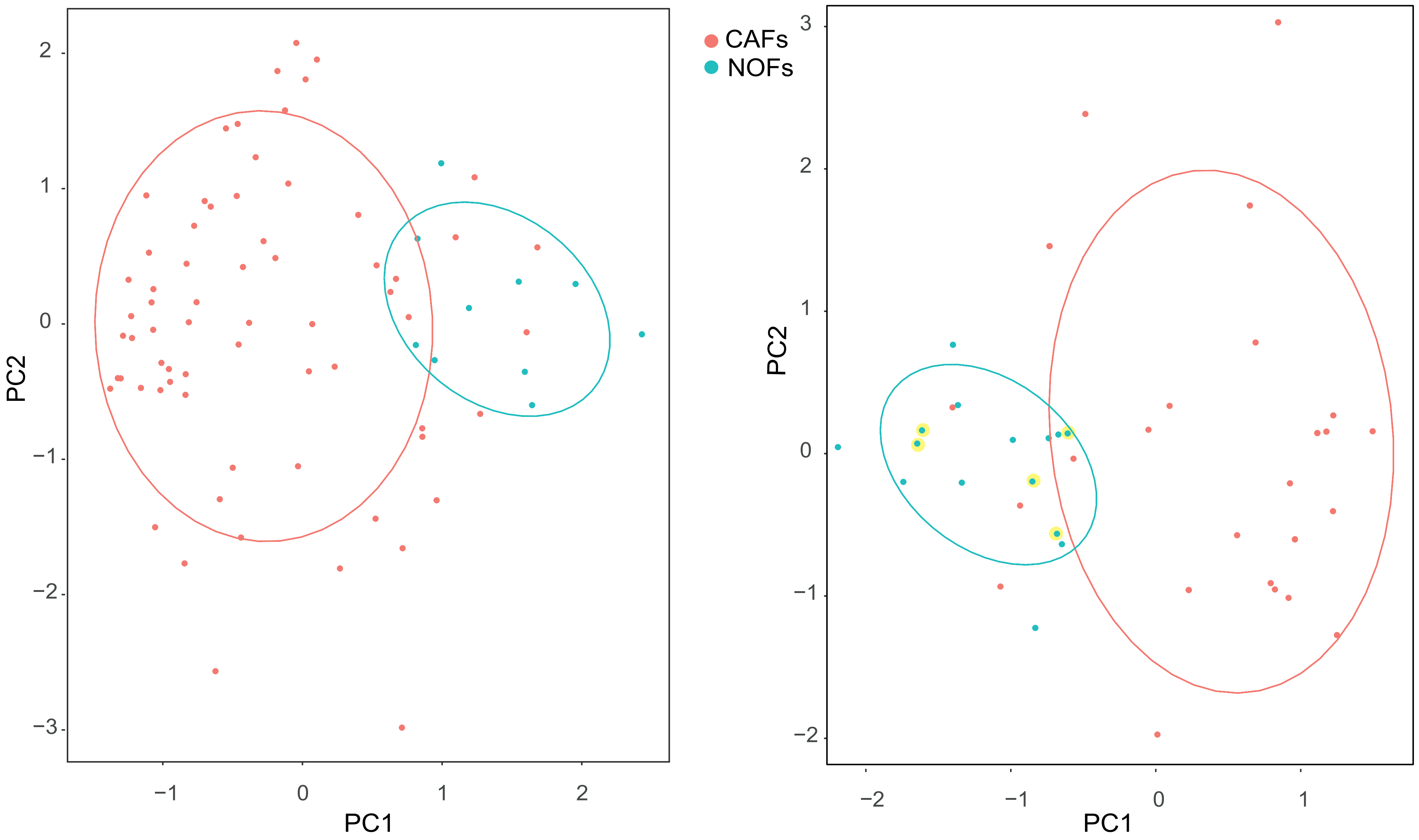

Supplementary Figure S2:

Heatmap representing the importance/contribution of 34 'stable' lncRNAs that identified to be differentially expressed in 50 iterations or more, in the Random Forest model accuracy. The black cells show iteration in which the corresponding lncRNA is not identified as being differentially expressed.

How the feature importance is measured?

The importance or relative contribution of each feature (differentially expressed lncRNA) in the Random Forest performance has been estimated based on the 'mean decrease accuracy' measure. Accordingly, the prediction error on the out-of-bag portion of the training data was first recorded for each tree in the forest. Then, to measure the importance of a lncRNA, the values of that lncRNA were permuted among the training data and the out-of-bag error is again computed on this perturbed data set. The importance score for the lncRNA was then computed by averaging the difference in out-of-bag error before and after the permutation over all trees.
The score is normalised by the standard deviation of these differences.

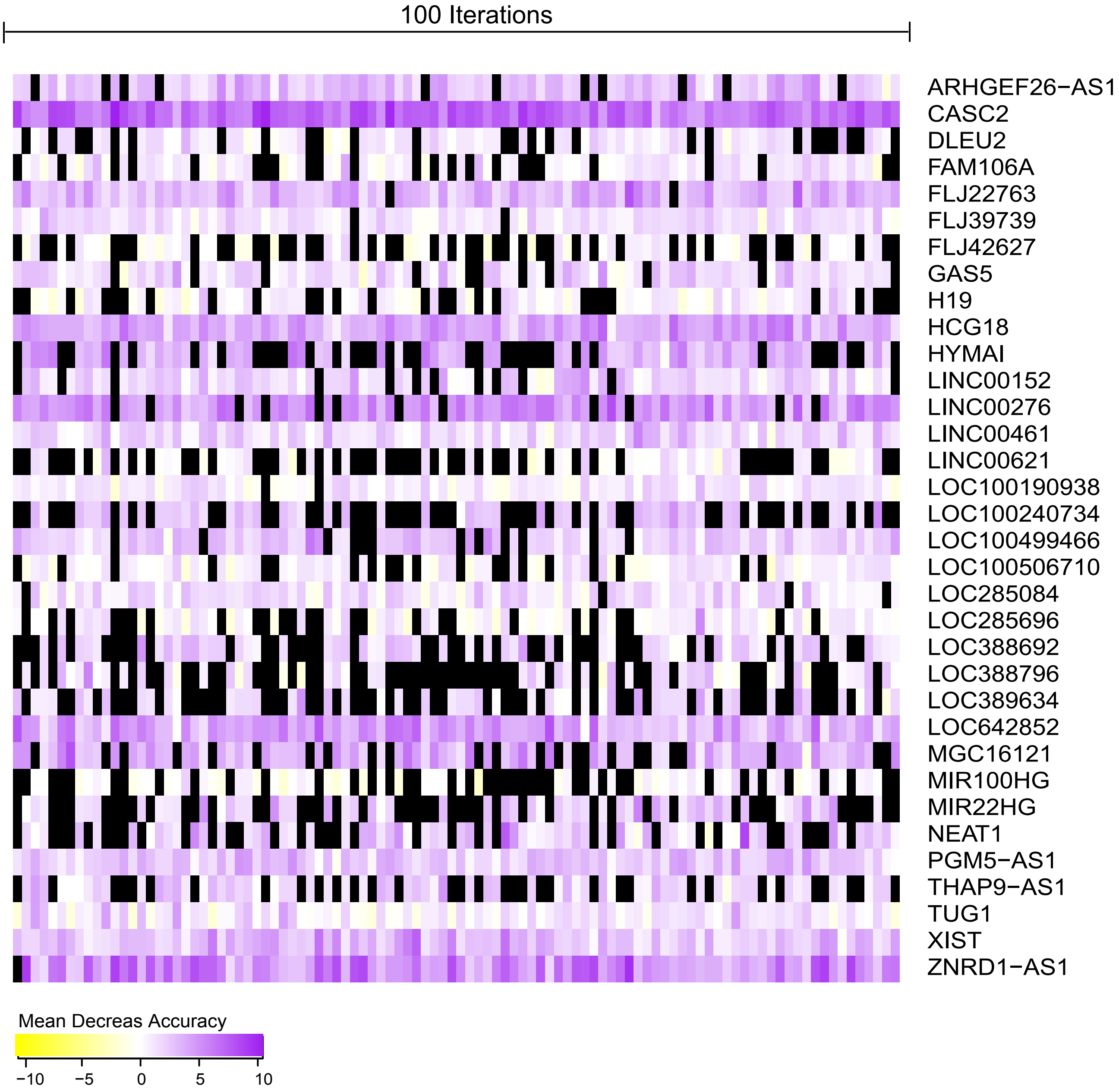

Supplementary Figure S3:

Visualization of the network of transcription factor – lncRNA and transcription factor – target gene interactions. Transcription factors are shown in yellow triangles, target genes in blue circles and lncRNAs in purple diamonds. In total, 1,266 interactions were identified and are depicted by the interconnecting grey lines.

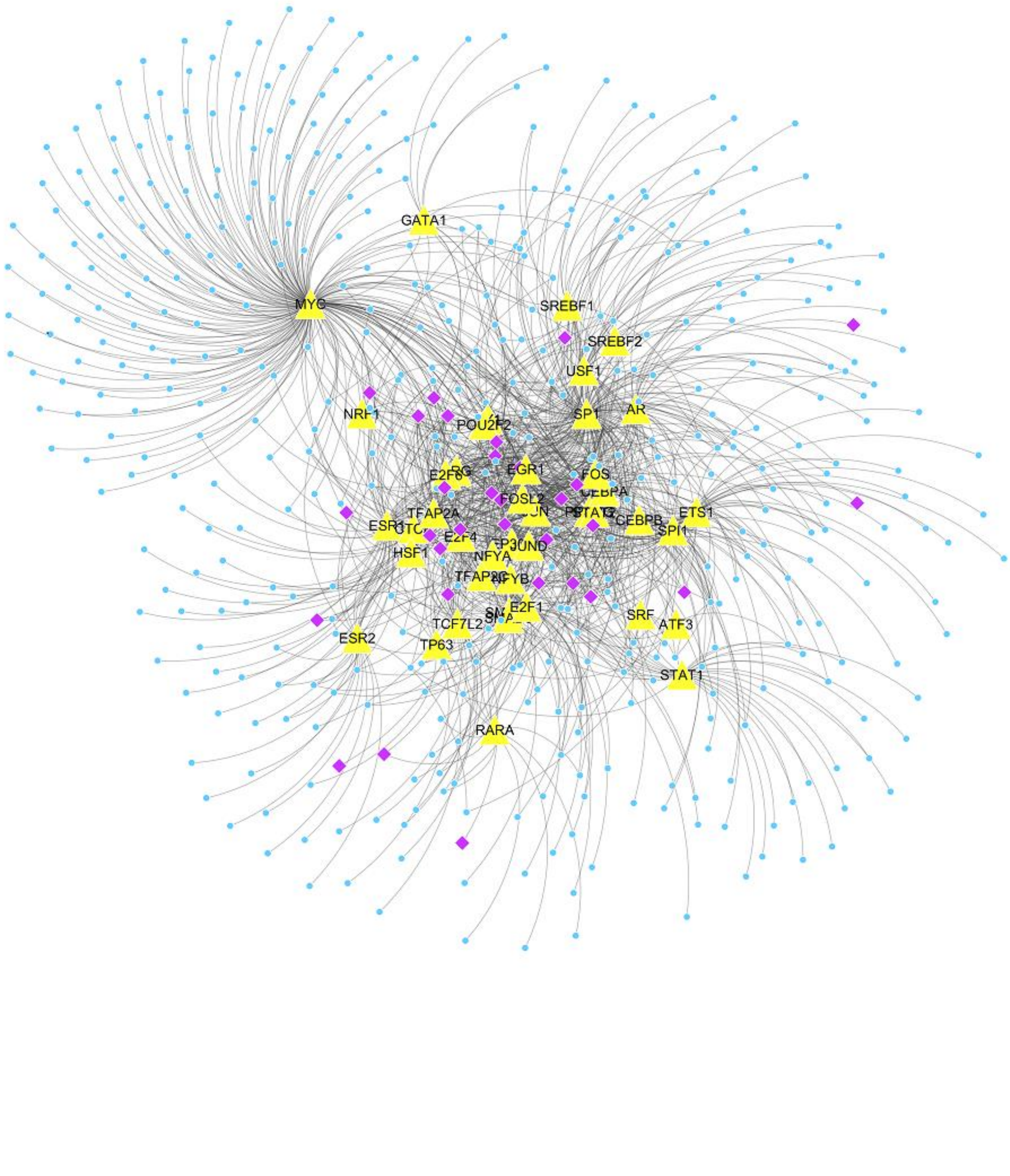

Supplement: Supplementary file 1 — Supplementary Information [file 41598_2017_10869_MOESM1_ESM.pdf]
